# Urban bat communities are affected by wetland size, quality, and pollution levels

**DOI:** 10.1002/ece3.2224

**Published:** 2016-06-16

**Authors:** Tanja Maria Straka, Pia Eloise Lentini, Linda Faye Lumsden, Brendan Anthony Wintle, Rodney van der Ree

**Affiliations:** ^1^ Australian Research Centre for Urban Ecology Royal Botanic Gardens Victoria, c/o School of BioSciences University of Melbourne Melbourne VIC Australia; ^2^ School of BioSciences University of Melbourne Melbourne VIC Australia; ^3^ Department of Environment, Land, Water and Planning Arthur Rylah Institute for Environmental Research 123 Brown Street Heidelberg VIC 3084 Australia

**Keywords:** Biodiversity, Chiroptera, conservation, heavy metals, light pollution, urbanization, wetland biota

## Abstract

Wetlands support unique biota and provide important ecosystem services. These services are highly threatened due to the rate of loss and relative rarity of wetlands in most landscapes, an issue that is exacerbated in highly modified urban environments. Despite this, critical ecological knowledge is currently lacking for many wetland‐dependent taxa, such as insectivorous bats, which can persist in urban areas if their habitats are managed appropriately. Here, we use a novel paired landscape approach to investigate the role of wetlands in urban bat conservation and examine local and landscape factors driving bat species richness and activity. We acoustically monitored bat activity at 58 urban wetlands and 35 nonwetland sites (ecologically similar sites without free‐standing water) in the greater Melbourne area, southeastern Australia. We analyzed bat species richness and activity patterns using generalized linear mixed‐effects models. We found that the presence of water in urban Melbourne was an important driver of bat species richness and activity at a landscape scale. Increasing distance to bushland and increasing levels of heavy metal pollution within the waterbody also negatively influenced bat richness and individual species activity. Areas with high levels of artificial night light had reduced bat species richness, and reduced activity for all species except those adapted to urban areas, such as the White‐striped free‐tailed bat (*Austronomus australis*). Increased surrounding tree cover and wetland size had a positive effect on bat species richness. Our findings indicate that wetlands form critical habitats for insectivorous bats in urban environments. Large, unlit, and unpolluted wetlands flanked by high tree cover in close proximity to bushland contribute most to the richness of the bat community. Our findings clarify the role of wetlands for insectivorous bats in urban areas and will also allow for the preservation, construction, and management of wetlands that maximize conservation outcomes for urban bats and possibly other wetland‐dependent and nocturnal fauna.

## Introduction

Wetlands are one of the most important and threatened ecosystems globally (Sala et al. 2000) and form key habitats in many environments including agricultural (Thiere et al. 2009), arid (Razgour et al. 2010), forested (Mensing et al. 1998), and urban areas (Smith and Chow‐Fraser 2010). Despite their importance for a wide array of species, wetlands continue to be cleared, drained, and modified. More than 50% of the world's wetlands have been destroyed over the past 100 years due to human activities (Dudgeon 2003), and today, global wetland cover is estimated to be 8–10 million km^2^ (6.2–7.6% of the Earth's land surface; Lehner and Döll 2004). Urbanization and the growing human population is arguably one of the most important threats to the persistence and quality of wetlands (Ehrenfeld 2000; Bassi et al. 2014) which has consequences for wetland‐dependent biota (Gibbs 2000).

Wetlands in human‐dominated environments are subject to numerous stressors such as pollution (Pettigrove and Hoffmann 2003; Göbel et al. 2007), alteration or removal of vegetation (Ehrenfeld 2000), and extreme fluctuations in water flow (Owen 1998). These activities can significantly impact on wetland‐dependent biota (Ehrenfeld 2000; Walsh et al. 2001). While most recent biodiversity research on urban wetlands has focused on waterbirds (DeLuca et al. 2004; Smith and Chow‐Fraser 2010), aquatic reptiles (Stokeld et al. 2014), amphibians (Hamer and McDonnell 2008), and invertebrates (Walsh et al. 2001; Carew et al. 2007), some taxa that benefit a great deal from wetlands, such as insectivorous bats, remain largely overlooked. To effectively manage wetlands for biodiversity conservation in urban areas, we need a better understanding of all the species that depend on these habitats as well as factors that determine suitability, occupancy, and persistence of biodiversity in wetlands at a range of spatial scales (Johnson et al. 2013).

Insectivorous bats are known to use wetlands extensively as feeding habitats in nonurban environments (Lloyd et al. 2006; Scott et al. 2010). Bat species that feed on smaller insects such as mosquitoes (Gonsalves et al. 2013) can even provide biological control of biting insects around water which may be particularly useful in urban environments. Some factors that benefit bats at nonurban wetlands include the presence of high‐quality riparian vegetation (Ober and Hayes 2008; Scott et al. 2010) and large wetland size (Razgour et al. 2010). Urban wetlands are often subject to changes in water quality, such as increased heavy metal pollution (Kellar et al. 2014) which can directly impact bat survival (Naidoo et al. 2013) and indirectly affect bats via changes to the aquatic invertebrate communities on which they feed (Walsh et al. 2001; Naidoo et al. 2013). The surrounding urban matrix can also influence insectivorous bats at urban waterways (Lintott et al. 2015), including the extent of bushland cover (Caryl et al. 2016; Threlfall et al. 2013) and degree of nocturnal artificial light (Mathews et al. 2015; Stone et al. 2015). Thus, to effectively manage urban wetlands for insectivorous bats, we need to understand the importance of these habitats as well as the key local‐ and landscape‐scale drivers that affect their suitability for bats.

Consequently, the aims of this study were to (1) investigate the importance of wetlands on bat species richness and individual species activity in an urban setting and (2) understand factors at landscape and individual wetland scales that shape bat communities at urban wetlands. We addressed these aims using data collected from 58 wetlands and 35 nonwetland habitat sites (which were ecologically similar sites without free‐standing water) across Melbourne in southeastern Australia. We discuss the implications of these findings in the context of understanding the impacts of urban design and provide tangible advice to planners and managers on how to maintain bat diversity through the conservation, management, and restoration of urban wetlands.

## Methods

### Study area and selection of wetlands

**Figure 1:**
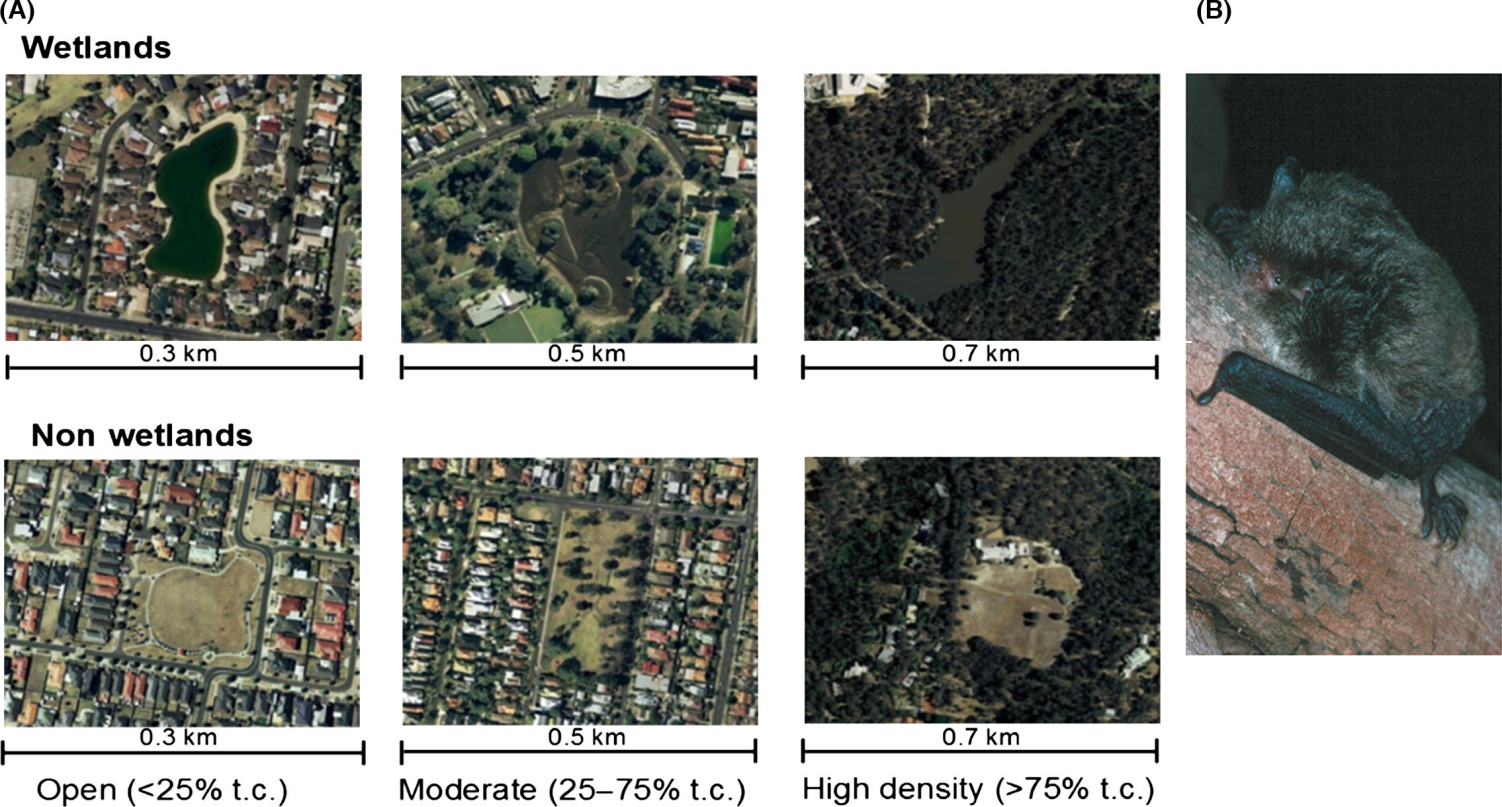
(A) Examples of wetland and nonwetland sites. Paired sites were similar with regard to the amount of area covered by standing water or open grassed area, tree cover (t.c.), and degree of urbanization. Source: Google Earth, retrieved July 2012. (B) Example of bat species occurring at wetlands in the Greater Melbourne area, the large‐footed myotis (*Myotis macropus*, photograph by L. Lumsden).

**Figure 2:**
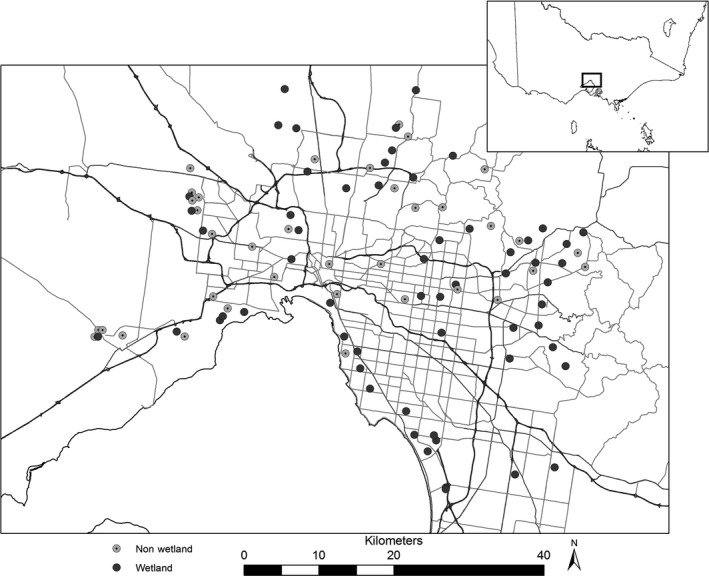
Distribution of study sites across greater Melbourne. Wetlands (*n* = 58) in dark gray; nonwetland habitats (*n* = 35) in pale gray. Only highways (dark gray lines) and major roads (pale gray lines) within Melbourne are shown to improve clarity. Inset shows the location of Melbourne within the State of Victoria, southeastern Australia.

This study was conducted within the greater metropolitan area of Melbourne (37°48′S, 144°55′E) in the state of Victoria, southeastern Australia. Melbourne is home to more than 4 million people and is one of the fastest growing capital cities in Australia, expected to reach 5.4 million inhabitants by 2031 (Department of Planning and Community Development 2012). Most of Melbourne's naturally occurring wetlands have been drained or filled‐in for development since European settlement in the mid‐1800s. Existing wetlands are predominantly those which have been constructed or modified to manage stormwater or for flood abatement (Melbourne Water 2010). We selected 58 standing bodies of water (i.e., wetlands, lakes, and ponds; hereafter referred to as “wetlands”) from an existing dataset of 120 water bodies used for a study of heavy metal pollutants (V. Pettigrove, University of Melbourne, pers. comm.). These 58 sites were stratified by wetland size (surface area), extent of surrounding tree cover within 5 m of the water's edge, and road density (highways and major roads) within a 1‐km buffer (which was used as a surrogate for urbanization). In order to test for the influence of water on insectivorous bats within the urban landscape, 35 of these 58 wetlands were paired with ecologically similar sites (in terms of tree cover and degree of urbanization). The only major difference between two sites in a pair (where minimum, average, and maximum distances between paired sites were 2, 4.6, and 23.6 km, respectively) was that instead of there being a water body, there was a cleared area of grassland at the reference site (Fig. 1). These reference sites are hereafter referred to as “nonwetland” sites. All sites were located in urban environments within 60 km of Melbourne's central business district (Fig. 2).

### Bat surveys and bat call analysis

Insectivorous bats were surveyed acoustically using Anabat detectors (SD1: Titley Electronics, Ballina, Australia) for two consecutive nights in summer (January and February) and two consecutive nights in autumn (March and April) in 2012 (*n* = 68 sites) or 2013 (*n* = 25 sites). Paired sites were surveyed simultaneously. One detector was positioned at the edge of each wetland or nonwetland site, in the area with the highest tree cover, and directed toward the center of the open water surface or open grassland area, respectively. All detectors were calibrated at deployment (Larson and Hayes 2000) and programmed to start recording 30 min before sunset and to stop recording 1 h after sunrise. Surveys were undertaken on mild nights (>10°C at sunset), without rain and with low wind speeds (≤small branches moving, which according to the Beaufort scale is a maximum wind speed of approx. 20–29 km/h). Data loggers (HOBO U23 Pro v2; Onset Computer Corporation Inc., Bourne, MA) attached to the bat detectors measured temperature at 15‐min intervals, from which the average nocturnal temperature was calculated for each survey night. Moon phase was noted for each night and classified as either a full moon (the night of the full moon, plus the four nights before and after the full moon) or a new moon (all other nights).

Recorded bat calls were analyzed using AnaScheme software and a regional identification key (Lumsden and Bennett 2005; Adams et al. 2010). Species identification was undertaken for bat passes with ≥ five calls that were identified with ≥50% certainty belonging to the same species (Lumsden and Bennett 2005). A bat pass is defined as the recording of a series of echolocation calls when a single bat passes the microphone (Abbott et al. 2009). In cases where one recording contained bat passes from two individuals, only one pass per recording was used. Bat call sequences which could not be identified to the species or genus level were grouped into “unknown” bat calls. Unknown bat calls were considered in the calculation of total bat activity. Manual checks using AnalookW software (C. Corben, www.hoarybat.com) were undertaken to confirm the calls of some species.

Species that could not be distinguished based on their echolocation calls were combined into species complexes: *Nyctophilus* spp. (*Nyctophilus geoffroyi* Leach, 1821 and *Nyctophilus gouldi* Tomes, 1858), *Scotorepens* spp. (*Scotorepens balstoni* [Thomas, 1906] and *Scotorepens orion* [Troughton, 1937]), and *Chalinolobus gouldii* (J. E. Gray, 1841)*/Mormopterus planiceps* (Peters 1866). *Nyctophilus geoffroyi* is more common in Melbourne than *N. gouldi*, with *N. geoffroyi* representing 85% of 1447 captures of *Nyctophilus* spp. in Melbourne (unpublished data DSE 2009); thus, most recorded calls within this complex are likely to belong to this species*. Scotorepens balstoni* and *S. orion* are both infrequently recorded in Melbourne (a total of 33 capture records, unpublished data DSE 2009). Although *C. gouldii* could frequently be identified using distinctive call characteristics, the call of this species sometimes overlaps with *M. planiceps*, and in these situations, the calls were recorded as a species complex. *Mormopterus planiceps* is rarely recorded in Melbourne and, when present, occurs predominantly in the northeast of the study area (unpublished data DSE 2009). Bat taxonomy and nomenclature follows Reardon et al. (2015).

### Landscape‐ and wetland‐scale variables

Five landscape‐scale measures of urbanization were calculated for all sites using ArcGIS version 10 (ESRI, Redlands, CA) within 500 m, 2 km and 5 km radii, namely (1) cover of impervious surfaces (buildings and roads); (2) number of 10 × 10 m cells containing woody vegetation greater than 2 m in height, and (3) level of nocturnal artificial light (visible‐near infrared [VNIR], see Table S1). We also measured the (4) straight‐line distance from the perimeter of each site to the nearest patch of remnant bushland >0.5 ha in size and (5) the distance to nearest other wetland or river (other than the one representing the wetland site). The size (ha) of each wetland and open area of each nonwetland site was calculated as its surface area. The cover of trees within 5 m of the water's edge or surrounding the open area was estimated on site at the time of the bat surveys and given a score of 0 to 1 (i.e., no trees to completely surrounded by trees, respectively).

Understorey vegetation, emergent aquatic vegetation, and water quality were measured at wetlands only. Understorey vegetation, defined as reeds and shrubs >50 cm in height surrounding the wetland within 5 m of its edge, was assessed similarly to tree cover on a scale of 0 to 1. The percentage of the whole water surface covered with emergent macrophytes was visually assessed and given a score of 0 to 1, corresponding with zero to 100% cover, respectively. The water quality of each wetland was assessed by calculating a sediment quality quotient (SQQ) following Stokeld et al. (2014) from a raw dataset of heavy metal levels (V. Pettigrove, University of Melbourne, pers. comm.), hereafter “sediment pollution”. High levels of SQQ are associated with sediment pollution that leads to degrading biological effects.

### Data analysis

Two sets of analyses, which corresponded to different spatial scales, were conducted in R v. 3.0.1 (R Development Core Team 2013). In the first set (hereafter “landscape models”), we used all the data from the 58 wetlands and 35 nonwetlands to investigate how wetland habitats influence bat species richness and activity (average number of bat passes per individual species over two sampling nights each season, controlling for a range of other landscape and site‐level variables). For the second set of models (hereafter “wetland models”), we investigated drivers of variation in bat species richness and activity at the 58 wetland sites only.

For both sets of analyses, we fitted generalized linear mixed‐effects models (GLMMs) with Poisson error distributions using a log link through the “lmer” function in the “lme4” package, with site and season (summer or autumn) fitted as random effects. As an initial step to avoid spatial correlation among landscape context variable (impervious surface, woody vegetation, and artificial light) taken at multiple scales, we identified the most appropriate scale (500 m, 2 km, or 5 km) by fitting single‐variable models to each variable at each scale and observing which scale provided the greatest reduction in residual deviance. For single‐variable models, this amounted to choosing the model, for any given species, with the lowest AIC across the three scales considered. The same approach was applied to choosing the appropriate vegetation variable (from onsite measurements at each wetland) for the wetland model. For correlated predictor variables (Pearson's correlations for all variables *r* < 0.2, *P* ≥ 0.05), the variable which led to the largest deviance reduction in a single‐variable model was retained for multivariable model fitting. GLMMs of individual species activity were run only for species that were present in at least 20% of sites (after Basham et al. 2011; see Table S2). We retained eight candidate variables for possible inclusion in both the multivariable landscape models and the wetland models, including the covariates moon and temperature that we thought were likely to influence bat activity (Table 1). The number of variables for the full candidate model of each species was limited to a maximum of one variable per 10 sites where it was recorded (Wintle et al. 2005) (see Table S2). All continuous predictors were centered and scaled prior to the analysis, and the variables “size,” “SQQ,” and “distance to bushland” were log‐transformed in order to improve convergence and aid parameter estimate interpretation. We used reverse stepwise variable reduction based on AICc (corrected for sample size) to arrive at a set of competitive landscape and wetland models for species richness, activity of each species, and total activity (Burnham and Anderson 2002, see Table S3). For each model, we calculated the predictive power (deviance reduction: [(null deviance−deviance of final model)/null deviance] × 100). We plotted the modeled effect and 95% confidence intervals of single variables on bat species richness and activity while holding all other variables constant at their mean, scaling bat activity to allow for comparison among species.

**Table 1:** Candidate variables tested in the models at the landscape scale and wetland scale

Variable name	Description	Landscape (all sites, *n *=* *93)	Wetland (only wetlands, *n *=* *58)
Site type	Wetland or nonwetland habitat	x	
Light	Visible‐near infrared (VNIR) radiance	x	x
Distance to bushland	Distance to the nearest bushland (>0.5 ha in size)	x	x
Distance to water	Distance to the nearest water source other than the site (wetland or river)	x	x
Tree cover	Proportion of tree cover within 5 m of the edge of the wetland or open area, scored from 0 to 1	x	x
Size	Surface area (ha) of wetland or open area of nonwetland habitat	x	x
SQQ	Sediment quality quotient, a measure of heavy metal pollution in wetland sediment		x
Temperature	Average nocturnal temperature	x	x
Moon	Full moon (nights of full moon incl. four days before and after) versus new moon phase (all other nights)	x	x

## Results

A total of 24,705 bat passes from 12 species of bats or species complexes were recorded from all sites, with 18,898 passes from 12 species of bats/complexes at the 58 wetland sites and 5807 passes from 11 species of bats/complexes at the 35 nonwetland sites. *Myotis macropus* was only recorded at wetland sites while remaining species or species complexes were recorded at wetland and nonwetland sites (see Table S2). All species or species complexes were recorded at enough of the sites (>20%) for modeling with the exception of *Mormopterus ridei* (Felten, 1964)*,* which was recorded at only 18% of all sites. The most frequently detected species were *Chalinolobus gouldii* (48.2% of recordings)*, Vespadelus vulturnus* (Thomas, 1914) (15.5%), and *Chalinolobus morio* (J. E. Gray, 1841) (10.2%). Nine species comprised <10% of recorded calls, with the least frequently detected species being *M. macropus* (Gould, 1855) (0.3%) and *M. ridei* (0.2%).

### Landscape‐scale drivers of bat species richness and individual species activity

The deviance reduction for our landscape‐scale models of bat species richness and individual species activity was between 15.2% (for *Miniopterus orianae oceanensis* [Maeda, 1982]) and 55.3% (for *Vespadelus darlingtoni* (Allen, 1933); Table 2). Presence of water was an important predictor for bat species richness, total bat activity, and nine of the 11 modeled species and species complexes (Table 2). Average nightly bat activity was 2.5 times higher at wetlands (mean = 136.6 passes) compared with nonwetland sites (mean = 52.5). Similarly, on average each wetland supported 1.1 more bat species than nonwetland sites (mean = 6.9 and 5.8, respectively).

**Table 2:** Parameter effect sizes (±SE) derived from Poisson GLMMs at the landscape scale (*n *=* *58 wetlands and *n *=* *35 nonwetland sites). Foraging space describes how cluttered the habitat is in which species forage. Distance to bushland and size were log‐transformed

Bat species/complex	Foraging space	Site type	Light	Distance to bushland	Distance to water	Tree cover	Size	Temp	Moon	Temp×moon	AICc	Deviance reduction
*Austronomus australis* (Aa)	Open	–	0.16 ± 0.24	−0.56 ± 0.26	0.41 ± 0.21	–	0.20 ± 0.22	−0.50 ± 0.12	0.53 ± 0.13	0.12 ± 0.13	1199.94	21.3%
*Chalinolobus gouldii* (Cg)	Uncluttered edge space	−0.66 ± 0.32	0.01 ± 0.15	−0.15 ± 0.15	−0.11 ± 0.15	0.44 ± 0.17	−0.13 ± 0.16	0.24 ± 0.03	−0.14 ± 0.04	−0.23 ± 0.04	3610.68	25.2%
*Chalinolobus gouldii/Mormopterus planiceps* (CgMp)	Uncluttered edge space	−0.34 ± 0.40	0.10 ± 0.19	–	–	0.21 ± 0.20	–	0.41 ± 0.16	−0.22 ± 0.24	−0.87 ± 0.29	881.27	22.6%
*Miniopterus orianae oceanensis* (Moo)	Uncluttered edge space	−1.61 ± 0.65	–	−0.99 ± 0.32	0.45 ± 0.29	–	–	–	−0.33 ± 0.18	–	542.06	15.2%
*Scotorepens* spp. (Sc)	Uncluttered edge space	−1.79 ± 0.44	−0.29 ± 0.19	−0.16 ± 0.21	–	0.86 ± 0.22	–	0.04 ± 0.13	−0.39 ± 0.16	0.41 ± 0.15	687.48	28.9%
*Chalinolobus morio* (Cm)	Cluttered edge space	−0.72 ± 0.45	−0.59 ± 0.22	−0.47 ± 0.23	–	–	–	0.46 ± 0.09	−0.72 ± 0.08	−0.89 ± 0.10	1413.52	25.6%
*Vespadelus darlingtoni* (Vd)	Cluttered edge space	−0.17 ± 0.93	−1.65 ± 0.46	−1.75 ± 0.52	–	–	–	0.01 ± 0.21	−1.96 ± 0.19	−0.67 ± 0.25	785.84	55.3%
*Vespadelus regulus* (Vr)	Cluttered edge space	–	−1.13 ± 0.49	–	–	–	–	–	−1.45 ± 0.21	–	457.00	30.0%
*Vespadelus vulturnus* (Vv)	Cluttered edge space	−0.97 ± 0.50	−0.52 ± 0.22	–	–	0.49 ± 0.26	–	0.33 ± 0.06	−0.44 ± 0.06	−0.76 ± 0.07	1704.81	28.2%
*Myotis macropus* (Mm)	Cluttered	Only at water	−0.82 ± 0.33	–	–	–	–	0.76 ± 0.40	−1.01 ± 0.38	−1.35 ± 0.50	214.92	23.0%
*Nyctophilus* spp. (Ny)	Cluttered	−1.02 ± 0.41	−0.40 ± 0.17	–	–	0.62 ± 0.20	–	0.49 ± 0.15	−0.36 ± 0.17	−0.71 ± 0.19	586.40	21.5%
Total bat activity		−0.96 ± 0.29	−0.21 ± 0.13	−0.25 ± .014	–	0.43 ± 0.15	–	0.18 ± 0.02	−0.37 ± 0.02	−0.31 ± 0.03	5090.05	29.2%
Bat species richness		−0.17 ± 0.10	−0.09 ± 0.04	−0.12 ± 0.05	–	0.13 ± 0.05	–	–	−0.11 ± 0.07	–	751.64	23.9%

**Figure 3:**
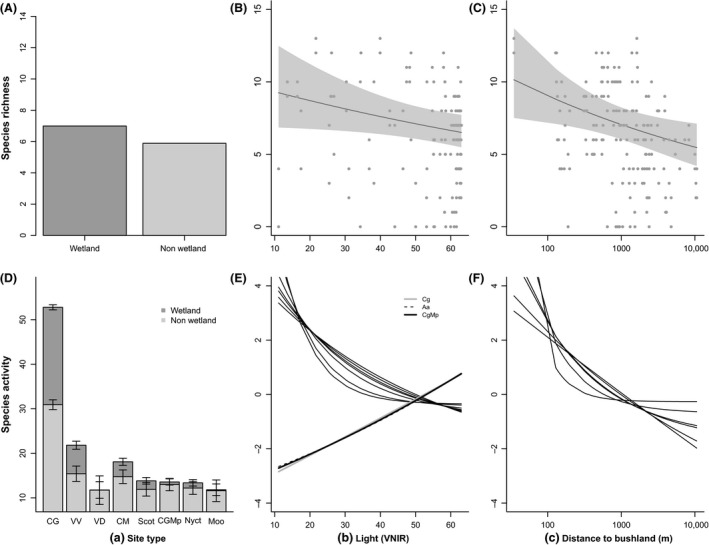
Top panel depicts the predicted relationship between mean bat species richness and landscape‐scale variables: (A) site type, (B) artificial light (VNIR), and (C) distance to the nearest bushland (m), with 95% confidence intervals shown in gray. The bottom panels (D–F) show the relationship between the average nightly activity of the individual species and these same variables when they were included in the final models, to indicate whether the responses of the species follow the broad species richness patterns. Predicted activity of each individual species was centered and scaled to allow for direct comparisons of the nature of the species' responses, however, because of this it is not possible to compare absolute predictions of activity among species. Patterns for some species are similar so individual lines may be obscured by others (e.g., Aa and CgMp in 3e). Species codes are listed in Tables 2 and 3.

Distance to the nearest patch of bushland and levels of artificial nocturnal light had the strongest effects on bat species richness and total bat activity at the landscape scale (Fig. 3, Table 2). Species‐specific responses to these predictors followed the same broad patterns, with the exception of the effect of light on *Austronomus australis* (J. E. Gray, 1838)*, C. gouldii,* and *C. gouldii/M. planiceps* (Fig. 3, Table 2). Bat species richness decreased from 10.1 to 5.5 species and average nightly bat activity from 177.3 to 39.1 bat passes as the distance to the nearest bushland increased from 34 m to 10,500 m. A strong negative response to this change in distance was found for *V. darlingtoni* in particular, with a decrease from 45.3 to 0 bat passes. As the relative level of artificial night light increased from 11 to 63 units (VNIR), bat species richness decreased from 9.2 species to 6.5 and average nightly bat activity from 251.6 to 116.8 bat passes. Light had the largest impact (effect size >1, Table 2) on the average nightly activity of the two *Vespadelus* species: *V. darlingtoni* (from 26.2 to 0.08 passes as the level of light increased) and *V. regulus* (Thomas, 1906) (from 5.6 to 0.05 passes). In contrast, we found a positive effect of light on average nightly activity of *A. australis* (0.66 to 1.1 passes as the level of light increased), *C. gouldii* (from 39.3 to 41.7 passes), and the *C. gouldii/M. planiceps* complex (from 1.5 to 2.2 passes).

**Table 3:** Parameter effect sizes (±SE) derived from Poisson GLMMs at the wetland scale (*n *=* *58 wetlands). Foraging space describes how cluttered the habitat is in which species forage. Distance to bushland, sediment quality quotient (SQQ), and size were log‐transformed

Bat species/complex	Functional guild	Light	Distance to bushland	Distance to water	Tree cover	SQQ	Size	Temp	Moon	Temp×moon	AICc	Deviance reduction
*Austronomus australis* (Aa)	Open	0.53 ± 0.38	−0.46 ± 0.40	–	–	−0.25 ± 0.37	0.32 ± 0.37	−0.65 ± 0.06	−0.41 ± 0.15	–	657.05	34.7%
*Chalinolobus gouldii* (*Cg*)	Uncluttered edge space	–	–	–	0.62 ± 0.16	−0.27 ± 0.17	–	0.49 ± 0.04	−0.28 ± 0.04	−0.33 ± 0.05	3255.30	10.2%
*Chalinolobus gouldii/Mormopterus planiceps* (CgMp)	Uncluttered edge space	−0.04 ± 0.18	−0.01 ± 0.20	–	0.26 ± 0.21	−0.46 ± 0.19	0.05 ± 0.19	0.11 ± 0.14	−0.04 ± 0.21	−0.20 ± 0.21	478.02	25.9%
*Miniopterus orianae oceanensis* (Moo)	Uncluttered edge space	–	−1.00 ± 0.44	–	–	–	0.56 ± 0.43	0.51 ± 0.29	−0.89 ± 0.25	−1.07 ± 0.34	415.74	13.0%
*Scotorepens* spp. (Sc)	Uncluttered edge space	−0.56 ± 0.26	0.02 ± 0.29	–	1.00 ± 0.31	−0.48 ± 0.28	–	−0.04 ± 0.14	−0.35 ± 0.18	0.52 ± 0.19	477.20	32.2%
*Chalinolobus morio* (Cm)	Cluttered edge space	−0.63 ± 0.31	−0.61 ± 0.32	–	–	–		0.48 ± 0.09	−0.84 ± 0.09	−1.13 ± 0.11	1000.16	29.9%
*Vespadelus darlingtoni* (Vd)	Cluttered edge space	−2.97 ± 0.95	−2.53 ± 1.00	–	–	–		1.71 ± 0.43	−3.66 ± 0.31	−3.29 ± 0.51	509.83	66.6%
*Vespadelus regulus* (Vr)	Cluttered edge space	−1.19 ± 0.51	–	–	–	−0.98 ± 0.55		–	−1.63 ± 0.23	–	260.61	45.3%
*Vespadelus vulturnus* (Vv)	Cluttered edge space	−0.47 ± 0.31	–	0.50 ± 0.29	–			0.07 ± 0.06	−0.32 ± 0.07	−0.60 ± 0.08	1088.02	33.4%
*Myotis macropus* (Mm)	Cluttered	−1.31 ± 0.48	–	–	–	−0.41 ± 0.65		–	–	–	109.01	27.9%
*Nyctophilus* spp. (Ny)	Cluttered	−0.30 ± 0.22	−0.31 ± 0.24	–	0.52 ± 0.25	–	–	0.35 ± 0.20	0.21 ± 0.23	−0.37 ± 0.25	370.53	25.0%
Total bat activity		−0.33 ± 0.19	−0.36 ± 0.19	–	0.39 ± 0.22	–		−0.07 ± 0.13	−0.32 ± 0.02	–	3583.00	32.3%
Bat species richness		−0.14 ± 0.06	−0.13 ± 0.06	–	0.18 ± 0.07	−0.13 ± 0.06	0.03 ± 0.06		−0.13 ± 0.10		457.53	28.0%

The distance to the next water source did not have a strong influence on bat species richness, but it was a strong positive predictor for the activity of *A. australis* and *M. orianae oceanensis* (Table 2). Tree cover was an important predictor for the activity of six species, total activity, and bat species richness, with the strongest effect found for *Scotorepens* spp. The surface area of open water or open grassland was a positive predictor for the large, fast‐flying *A. australis*, but negative for the smaller, more maneuverable *V. darlingtoni*. Activity of most bat species increased with ambient temperature and decreased during the full moon period, except for *A. australis* whose activity decreased with ambient temperature and increased during the full moon phase. Furthermore, we found an interaction between temperature and moon phase for most species (Table 2), while the reverse of this interaction was found for *A. australis*. *Scotorepens* spp. did not follow any of these patterns.

### Wetland factors driving bat species richness and species‐specific responses

**Figure 4:**
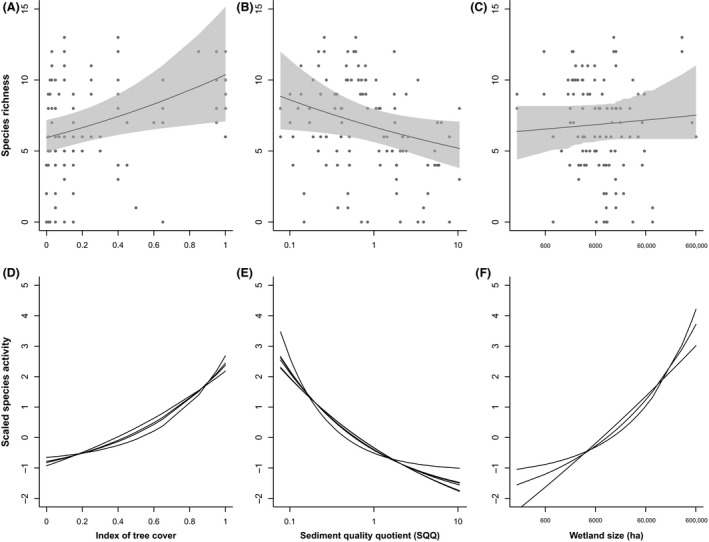
Top panel depicts the predicted relationship between mean bat species richness and wetland‐scale variables: (A) an index of tree cover, (B) sediment quality quotient, SQQ, and (C) wetland size (ha), with 95% confidence intervals shown in gray. The bottom panels (D–F) show the relationship between the average nightly activity of the individual species where they were included in the final models for that species to indicate whether the responses of the species follow the broad species richness patterns. Predicted activity of each individual species was centered and scaled to allow for direct comparisons of the nature of the species' responses, however, because of this it is not possible to compare absolute predictions of activity between species.

The deviance reduction for wetland models ranged from 10.2% (for *C. gouldii*) to 66.6% (for *V. darlingtoni,* Table 3). Tree cover and sediment pollution had the strongest effects on bat species richness at the wetland scale (Table 3). Species‐specific responses generally followed the same broad patterns as overall richness and total activity. Bat species richness increased from 5.9 to 10.4 species as tree cover increased from 0 (no trees) to 1 (completely surrounded by trees). *Chalinolobus gouldii* and *Scotorepens* spp., which typically forage in spaces around vegetation, showed the greatest positive response to tree cover at the wetland scale (23.7 to 155.9 passes and 0.7 to 15.3 passes with increased tree cover, respectively). Bat species richness decreased from 8.8 to 5.2 as sediment pollution increased from SQQ levels 0 to 10.43. The species most affected by sediment pollution were *V. regulus* (activity decreased from 1.9 to 0.03 passes per night with increasing level of SQQ) and *M. macropus* (0.05 to 0.01 passes). Wetland size was also a contributing factor but had a comparatively weaker effect (Fig. 4). As wetland size increased from 0.016 ha to 60.56 ha, bat species increased from 6.4 to 7.5 species, with *M. orianae oceanensis* being the most positively affected (increase in activity from 0.07 to 1.6 passes per night).

## Discussion

Urban wetlands and the fauna that they support provide important ecosystem services to society, but are at risk of loss or degradation through anthropogenic activities. We have shown that presence of water, levels of artificial nocturnal light, and the proximity to bushland are important landscape‐scale drivers of the richness and activity of insectivorous bats. Wetlands that supported the most bat activity and species‐rich assemblages were large and had high levels of tree cover and low levels of sediment pollution.

### Landscape drivers of variation in bat richness and activity

Our results suggest that wetlands are important for insectivorous bats in urban environments, with greater activity and higher species richness than nonwetland habitats. *Myotis macropus,* a species adapted to foraging over water, was found at a quarter of the wetland sites but none of the nonwetland sites, with levels of artificial nocturnal light and sediment pollution being key factors influencing its activity. In addition, the occurrence of *M. macropus* may be influenced by its reliance on large roost trees located near waterways (Campbell 2009), many of which have been extensively cleared from urban areas (Le Roux et al. 2014).

The proximity of wetlands to patches of natural bushland was the strongest landscape‐scale predictor of bat species richness and activity. Many bats in Australia are strongly associated with native forests and woodlands for foraging and roosting (Churchill 2008), including those in urban environments (Basham et al. 2011). Bats also require a range of complementary habitats to occur within their home ranges, to provide roosts, and feeding and drinking grounds. This is especially critical for species with relatively small home ranges (Lookingbill et al. 2010) such as *V. darlingtoni,* which selects large trees for roosting (Herr and Klomp 1999) and was the species most affected by distance to bushland in our study. Some species have also been found to occupy smaller home ranges in urban environments (Threlfall et al. 2013). Therefore, protecting and restoring bushland near waterways and also creating new wetlands in close proximity to existing bushland will be beneficial for bat conservation, particularly for species with small home ranges.

Artificial nocturnal light has harmful effects on biodiversity (Hölker et al. 2010; Blackwell et al. 2015). There has been increasing awareness of the effects of artificial nocturnal light on bats in recent years, and research suggests that responses are species‐specific (Mathews et al. 2015; Stone et al. 2015). Broad‐winged and slow‐flying bats that typically forage in cluttered areas and avoid open areas tend to be more sensitive to light (Rydell 1991; Stone et al. 2009). This was supported by our findings. The *Nyctophilus* spp. complex was less active in highly lit areas, as was the case in two previous studies of *N. gouldi* (Haddock 2008; Threlfall et al. 2013). High levels of artificial nocturnal light also had a negative influence on two other small forest bat species (*V. regulus* and in particular on *V. darlingtoni*), and overall negatively affected bat species richness and activity. In contrast, three species that tend to fly in more open areas, *A. australis, C. gouldii,* and *C. gouldii/M. planiceps* responded positively to light, possibly because of the insect‐rich food sources that streetlights provide (Rydell 1992; Avila‐Flores and Fenton 2005). However, increased activity around lighting sources may increase risk of predation (Stone et al. 2009). Approximately 75% of the 450,000 streetlights in Melbourne are mercury vapor (Equipment Energy Efficiency Program 2011) which are known to attract high numbers of insects (Rydell 2006). The predominantly negative effect of artificial light on most bat species in our study suggests that lights are having damaging effects on bat habitats, although it is not clear whether other types of lighting would be more benign. Finding appropriate lighting solutions may improve conditions for urban bats, and this remains a research priority.

### Wetland factors driving bat species richness and species‐specific responses

Heavy metals carried by urban storm water runoff contribute to the degradation of urban water (Göbel et al. 2007). There is a clear link between the level of heavy metal pollution in wetland sediments and the amount of impervious surface in the surrounding landscape (Pettigrove and Hoffmann 2003), and for this reason, heavy metal pollution is likely to be particularly problematic for urban wetlands. This is the first study to look at the impacts of heavy metal pollution on insectivorous bats associated with urbanization. Past studies have shown that heavy metal pollution can negatively affect the richness of nocturnal flying insects around wetlands (Naidoo et al. 2013; T. M. Straka et al. unpublished data). Because these are important prey items for bats, these effects may flow‐on in the form of negative secondary responses. This is supported by our finding that *M. macropus*, an aquatic specialist that feeds predominantly on aquatic insects (Campbell 2009), and *V. regulus* which regularly feeds on Dipterans (Lumsden and Bennett 2005) were the two species most negatively affected by sediment pollution. This also aligns with findings that the activity of *M. macropus* was reduced at contaminated coastal wetlands (Clarke‐Wood 2013). However, given that some aquatic invertebrates are tolerant to contamination (Carew et al. 2007), heavy metal pollution does not necessarily mean fewer prey items for all bat species. A study in South Africa found toxic metals in the tissues of trawling banana bats (*Neoromicia nanus*) that exploit swarming pollution‐tolerant midges at wastewater‐polluted sites (Naidoo et al. 2013). While an acceptable or safe level of accumulated heavy metals in bats is unknown, it is likely that thresholds exist, beyond which critical long‐term effects may occur.

Trees around wetlands provide critical resources for bats, including roosts (Campbell 2009), landmarks for orientation (Limpens and Kapteyn 1991), shelter from wind and predators (Verboom and Spoelstra 1999), habitats that support higher insect densities (e.g., Fukui et al. 2006; Carew et al. 2007), and shade over the water surface for species which are light‐sensitive (Rydell et al. 1996). For this reason, it was not surprising that wetland sites with greater tree cover around their perimeters supported higher bat species richness and activity of species which typically forage close to vegetation and in gaps between trees such as *C. gouldii* and *Scotorepens* spp. In some cases, high densities of trees around very small wetlands may limit their suitability for less‐maneuverable species of bats (Ciechanowski 2002), but this was not apparent in our study.

We did not explore the extent to which bats were using wetlands for foraging versus drinking, because recordings of feeding and drinking buzzes are indistinguishable when recorded with Anabat detectors (Griffiths 2013). However, the number of feeding/drinking buzzes has been shown to correlate broadly with overall numbers of bat passes (e.g., Law et al. 1998; O'Donnell 2000), and aquatic habitats are known to form important foraging grounds for insectivorous bats (Fukui et al. 2006). Although some of the activity recorded in our study may have resulted from bats commuting through the wetlands, this is likely to represent only a small proportion of recorded passes, and we assume that most activity at wetlands was likely due to bats' foraging or drinking. We recorded the same species and species complexes of bats at wetlands and nonwetland habitats as an earlier study across the greater Melbourne region, with the exception of one species: *Falsistrellus tasmaniensis* (Caryl et al. 2016). Although our study showed the benefit of wetlands compared with nonwetland habitats for insectivorous bats in an urban environment, it is important to bear in mind that bats still benefit from green spaces in urban areas irrespective of whether water is present or not (Caryl et al. 2016; Threlfall et al. 2011). Nevertheless, wetlands generally provide high‐quality habitats for insectivorous bats in urban environments due to their highly productive nature; thus, they warrant increased attention in order to improve bat conservation outcomes within the urban matrix.

## Conclusions

Wetlands are important habitats for insectivorous bats in urban areas and influence both species richness and activity. Wetlands that support high bat diversity are those which are in close proximity to a bushland, and can be improved by planting trees around wetlands to provide roosting and foraging habitat as well as shade from artificial lighting. The habitat for bat species that avoid lights could be improved by removing unnecessary lighting, reducing the intensity of required lighting and by limiting spillover by modifying the design of lighting fixtures (Threlfall et al. 2013; Blackwell et al. 2015). Further research into the benefits of changing lighting type is required. Disconnecting and/or slowing the direct flow of stormwater into existing wetlands, and ensuring new wetlands remain disconnected through water‐sensitive urban design (Donofrio et al. 2009) will reduce the amount of heavy metals accumulating in sediments and benefit insectivorous bats. A more complete understanding of the role and functioning of all types of water bodies, including rivers and streams, in urban landscapes for bats would be highly beneficial in designing and managing rapidly urbanizing areas. These findings are likely to be relevant for other nocturnal and aquatic‐dependent taxa.

## Data Accessibility

Raw data and R scripts have been deposited to the Harvard Dataverse and are accessible under doi: http://dx.doi.org/10.7910/DVN/MJF2D5.

## Conflict of Interest

None declared.

## Supporting information


**Table S1.** Landscape‐scale measures of urbanization.
**Table S2.** The number of calls from insectivorous bats recorded at 93 sites (58 wetlands and 35 non‐wetland habitat sites).
**Table S3.** Model selection results for landscape and wetland models.
