# Supplementary material for: Urban bat communities are affected by wetland size, quality, and pollution levels
Source: Ecol Evol. 2016 Jun 16;6(14):4761–74. doi: 10.1002/ece3.2224 (PMC4979705; doi:10.1002/ece3.2224)
Supplement: Supplementary file 1 — Table S1. Landscape‐scale measures of urbanization. Table S2. The number of calls from insectivorous bats recorded at 93 sites (58 wetlands and 35 non‐wetland habitat sites). Table S3. Model selection results for landscape and wetland models. [file ECE3-6-4761-s001.docx]

# **Online Supporting Information**

**Table S1. Landscape-scale measures of urbanisation.**

| Layer name | Data type | Source | Description | Resolution |
| --- | --- | --- | --- | --- |
| Impervious surface | Vector | Direct connected imperviousness (DCI); unpublished data, Melbourne Water | Proportional cover of impervious surfaces (roads and houses) | 1:25,000 |
| Trees | Raster  (binary) | Department of Environment and Primary Industries 2006, SPOT Panchromatic imagery | Presence/absence grid of woody vegetation greater than 2 m in height | 1:25,000, 10 m pixel size |
| Light | Raster  (continuous) | NOAA (2012) Earth observation group: Version 4 dmsp-ols night-time lights time series. Accessed 29 July, 2014, URL <http://ngdc.noaa.gov/eog/dmsp/downloadV4composites.html> | Visible-near infrared (VNIR) radiance (quantity of radiation that passes through or is emitted from a surface). Relative measurement. | 1 km pixel size |
| Distance to the nearest bushland | Vector | EVC data (native vegetation); Department of Environment and Primary Industries, 2009 | Straight-line distance (m) | - |
| Distance to the nearest water source | Vector | Hydrological features; Department of Environment and Primary Industries, 2009 | Straight-line distance (m) | - |

## Table S2. The number of calls from insectivorous bats recorded at 93 sites (58 wetlands and 35 non-wetland habitat sites).

|  | Total number of calls (% within wetland, % within non-wetland) | Sites recorded (%) |
| --- | --- | --- |
| *Chalinolobus gouldii* | 11286 (73 %, 27 %) | 93 (100%) |
| *Vespadelus vulturnus* | 3628 (73 %, 27 %) | 85 (91%) |
| *Chalinolobus morio* | 2387 (80 %, 20 %) | 79 (85%) |
| *Vespadelus darlingtoni* | 2145 (96 %, 4 %) | 40 (43%) |
| *Austronomus australis* | 1268 (78 %, 22 %) | 77 (83%) |
| *Scotorepens* spp*.* | 756 (85 %, 15 %) | 63 (68%) |
| *C. gouldii /Mormopterus planiceps* | 722 (58 %, 42 %) | 77 (83%) |
| *Vespadelus regulus* | 436 (75 %, 25 %) | 34 (37%) |
| *Nyctophilus* spp*.* | 427 (71 %, 29 %) | 64 (69%) |
| *Miniopterus orianae oceanensis* | 263 (82 %, 18 %) | 37 (40%) |
| *Myotis macropus* | 74 (100 %, 0 %) | 24 (26%) |
| *Mormopterus ridei* | 47 (91 %, 9 %) | 17 (18%) |

**Table S3. Model selection results for landscape and wetland models.**

Model selection results for generalised linear mixed effects models (GLMMs) with Poisson error distributions and a log link. Reverse stepwise variable reduction based on AICc (corrected for sample size). Δ AICc = AICc – minimum (AICc). Models shown within Δ AICc < 4. Variable terms: site type = wetland or non-wetland, light = relative artificial light within 5 km buffer, bush = distance to next bushland, water = distance to next water body (wetland, lake or river), trees = tree coverage around wetland border or grassy area, SQQ = sediment quality quotient (heavy metal pollution), size = area of wetland or grassy area, temp*moon = Interaction between temperature and moon phase. Distance to bushland, SQQ and size were log transformed.

*Landscape-scale models (n = 58 wetlands and n = 35 non-wetland sites)*

| **Response** | **Model #** | **Site type** | **Light** | **Bush** | **Water** | **Trees** | **Size** | **Moon** | **Temp* Moon** | **AICc** | **Δ AICc** | **Akaike weight** |
| --- | --- | --- | --- | --- | --- | --- | --- | --- | --- | --- | --- | --- |
| Species richness | 3 | x | x | x |  | x |  | x |  | 751.64 | 0.00 | 0.66 |
|  | 2 | x | x | x |  | x | x | x |  | 753.70 | 2.06 | 0.24 |
|  | 1 | x | x | x |  | x | x |  | x | 755.94 | > 4 | 0.97 |
|  | Full | x | x | x | x | x | x |  | x | 757.98 | > 4 | 1 |
| Bat activity models | 2 | x | x | x |  | x |  |  | x | 5090.05 | 0.00 | 0.68 |
|  | 1 | x | x | x |  | x | x |  | x | 5092.30 | 2.25 | 0.22 |
|  | Full | x | x | x | x | x | x |  | x | 5094.05 | 4.00 | 1.00 |
| *Chalinolobus gouldii* ^a^ | Full | x | x | x | x | x | x |  | x | 3610.68 | 0.00 | 1 |
| *Vespadelus vulturnus* | 3 | x | x |  |  | x |  |  | x | 1704.81 | 0.00 | 0.62 |
|  | 2 | x | x |  | x | x |  |  | x | 1706.73 | 1.92 | 0.24 |
|  | 1 | x | x | x | x | x |  |  | x | 1708.41 | 3.60 | 0.10 |
|  | Full | x | x | x | x | x | x |  | x | 1710.27 | > 4 | 0.04 |
| *Vespadelus darlingtoni* ^a^ | Full | x | x | x |  |  |  |  | x | 785.84 | 0.00 | 1 |
| *Chalinolobus morio* | 3 | x | x | x |  |  |  |  | x | 1413.52 | 0.00 | 0.37 |
|  | 2 | x | x | x |  |  | x |  | x | 1413.76 | 0.23 | 0.33 |
|  | 1 | x | x | x |  | x | x |  | x | 1414.62 | 1.09 | 0.21 |
|  | full | x | x | x | x | x | x |  | x | 1416.41 | 2.88 | 0.09 |
| *Austronomus australis* | 2 |  | x | x | x |  | x |  | x | 1199.94 | 0.00 | 0.70 |
|  | 1 |  | x | x | x | x | x |  | x | 1202.22 | 2.28 | 0.23 |
|  | Full | x | x | x | x | x | x |  | x | 1204.56 | > 4 | 1.00 |
| *Scotorepens* spp*.*^a^ | Full | x | x | x |  | x |  |  | x | 687.48 | 0.00 | 1 |
| *C. gouldii/Mormopterus planiceps* | 3 | x | x | x |  | x |  |  | x | 881.27 | 0.00 | 0.64 |
|  | 2 | x | x | x |  | x |  |  | x | 883.12 | 1.90 | 0.25 |
|  | 1 | x | x |  | x | x | x |  | x | 885.31 | > 4 | 0.08 |
|  | Full | x | x | x | x | x | x |  | x | 887.66 | > 4 | 0.26 |
| *Vespadelus regulus* | 1 |  | x |  |  |  |  | x |  | 457.00 | 0.00 | 0.65 |
|  | Full | x | x |  |  |  |  | x |  | 458.24 | 1.24 | 0.35 |
| *Nyctophilus* spp*.*^a^ | Full | x | x |  |  | x |  |  | x | 586.40 | 0.00 | 1 |
| *M. orianae oceanensis* ^a^ | Full | x |  | x | x |  |  | x |  | 542.06 | 0.00 | 1 |
| *Myotis macropus* ^a^ | Full |  | x |  |  |  |  |  | x | 214.92 | 0.00 | 0.91 |

^a^ Other models for bat species were Δ AIC > 4, therefore only the best model is presented.

*Wetland-scale models (n = 58 wetlands)*

| **Response** | **Model #** | **SQQ** | **Light** | **Bush** | **Water** | **Trees** | **Size** | **Moon** | **Temp** | **Temp* Moon** | **AICc** | **Δ AICc** | **Akaike weight** |
| --- | --- | --- | --- | --- | --- | --- | --- | --- | --- | --- | --- | --- | --- |
| Species richness | 2 | x | x | x |  | x | x | x |  |  | 457.53 | 0.00 | 0.64 |
|  | 1 | x | x | x | x | x | x | x |  |  | 458.93 | 1.40 | 0.31 |
|  | Full | x | x | x | x | x | x |  |  | x |  | > 4.00 |  |
| Activity | 3 |  | x | x |  | x |  |  |  | x | 3583.00 | 0.00 | 0.58 |
|  | 2 |  | x | x | x | x |  |  |  | x | 3584.80 | 1.81 | 0.24 |
|  | 1 | x | x | x | x | x |  |  |  | x | 3585.97 | 2.97 | 0.13 |
|  | Full | x | x | x | x | x | x |  |  | x |  | > 4.00 |  |
| *Chalinolobus gouldii* | 3 | x |  |  |  | x |  |  |  | x | 3255.30 | 0.00 | 0.29 |
|  | 4 |  |  | x |  | x |  |  |  | x | 3255.56 | 0.26 | 0.25 |
|  | 2 | x |  | x |  | x |  |  |  | x | 3255.94 | 0.64 | 0.21 |
|  | 1 | x |  | x |  | x | x |  |  |  | 3256.10 | 0.80 | 0.19 |
|  | Full | x |  | x | x | x | x |  |  | x | 3258.55 | 3.24 | 0.06 |
| *Vespadelus vulturnus* | 3 |  | x |  | x |  |  |  |  | x | 1088.02 | 0.00 | 0.49 |
|  | 2 | x | x |  | x |  |  |  |  | x | 1088.67 | 0.65 | 0.35 |
|  | 1 | x | x |  | x | x |  |  |  | x | 1090.78 | 2.77 | 0.12 |
|  | Full | x | x | x | x | x |  |  |  | x |  | > 4.00 |  |
| *Vespadelus darlingtoni* | 2 |  | x | x |  |  |  |  |  | x | 509.83 | 0.00 | 0.60 |
|  | 1 | x | x | x |  |  |  |  |  | x | 511.49 | 1.66 | 0.26 |
|  | Full | x | x | x |  | x |  |  |  | x | 512.86 | 3.03 | 0.13 |
| *Chalinolobus morio* | 2 |  | x | x |  |  |  |  |  | x | 1000.16 | 0.00 | 0.50 |
|  | 1 |  | x | x |  | x |  |  |  | x | 1001.01 | 0.85 | 0.33 |
|  | Full |  | x | x |  | x | x |  |  | x | 1002.25 | 2.09 | 0.18 |
| *Austronomus australis* | 2 | x | x | x |  |  | x | x | x |  | 657.05 | 0.00 | 0.70 |
|  | 1 | x | x | x |  | x | x | x | x |  | 658.72 | 1.68 | 0.30 |
|  | Full | x | x | x |  | x | x |  |  | x |  | > 4.00 |  |
| *Scotorepens* spp*.* | 1 | x | x | x |  | x |  |  |  | x | 477.20 | 0.00 | 0.60 |
|  | Full | x | x | x |  | x | x |  |  | x | 478.02 | 0.81 | 0.40 |
| *C. gouldii/ Mormopterus planiceps* ^a^ | Full | x | x | x |  | x | x |  |  | x | 478.02 | 0.00 | 1 |
| *Vespadelus regulus* | 1 | x | x |  |  |  |  | x |  |  | 260.61 | 0.00 | 0.88 |
|  | Full | x | x |  |  |  |  |  |  | x |  | >4.00 |  |
| *Nyctophilus* spp*.* | 2 | x | x | x |  | x |  |  |  | x | 370.53 | 0.00 | 0.51 |
|  | 1 |  | x | x |  | x | x |  |  | x | 371.25 | 0.72 | 0.36 |
|  | Full | x | x | x |  | x | x |  |  | x | 373.32 | 2.79 | 0.13 |
| *M. orianae oceanensis* | 1 |  |  | x |  |  | x |  |  | x | 415.74 | 0.00 | 0.76 |
|  | Full |  |  | x |  | x | x |  |  | x | 418.03 | 2.30 | 0.76 |
| *Myotis macropus* ^a^ | Full | x | x |  |  |  |  |  |  |  | 109.01 | 0.00 | 1 |

^a^ Other models for bat species were Δ AIC > 4, therefore only the best model is presented.
